# Robust prediction of response to growth hormone therapy by five PBMC-based transcripts

**DOI:** 10.1016/j.gendis.2026.102132

**Published:** 2026-03-06

**Authors:** Valerie van Kekem, Irene Victoria Bermúdez-Pérez, Alexandra E. Kulle, Maik Welzel, Nadine Hornig, Gerhard Binder, Matthias Hübenthal, Robert Häsler, Paul-Martin Holterhus

**Affiliations:** a Department of Neurology, University Hospital of Schleswig-Holstein, UKSH, Campus Kiel, Arnold-Heller-Straße 3, Kiel 24105, Germany; b Department of Dermatology and Allergy, University Hospital of Schleswig-Holstein, UKSH, Campus Kiel, Arnold-Heller-Straße 3, Kiel 24105, Germany; c Department of Pediatrics I, Pediatric Endocrinology and Diabetes, University Hospital of Schleswig-Holstein, UKSH, Campus Kiel, Kiel 24105, Germany; d Institute of Clinical Chemistry, UKSH, Kiel/Lübeck 24105, Germany; e Private Practice of Pediatrics, Am Ochsenkopf 1, Eckernförde 24340, Germany; f Institute of Human Genetics, University Hospital of Schleswig-Holstein, UKSH, Campus Kiel, Kiel 24105, Germany; g Pediatric Endocrinology, University Children’s Hospital Tübingen, Tübingen 72076, Germany

Short stature affects about 150 million children worldwide and results in lifelong consequences, including reduced bone mineral density, increased psychosocial stress, and lower quality of life. Since the approval of recombinant human growth hormone (r-hGH) in 1987, its use in children with short stature has expanded across multiple diagnoses.^1^ Although serious side effects are uncommon, the therapy is costly and burdensome because it requires daily injections and sustained adherence over years. Individual treatment responses vary considerably, and various prediction approaches have been proposed to support long-term management. Initial predictions relied on auxological, biochemical, radiological, and demographic data, while more recent models incorporated molecular data such as single nucleotide polymorphisms and transcriptome profiles^2^, ^3^, ^4^. Despite their relevance, these predictions are not in clinical routine use because of low accuracy, complex workflows, and the need to measure hundreds of features. To address these limitations, we aimed to develop a prediction model with i) high accuracy, ii) a workflow embedded in established routine short stature diagnostics, and iii) a small set of predictive features.

Seventy-one patients with growth hormone deficiency, bioinactive growth hormone, small for gestational age, or Turner syndrome were enrolled in this single-center study (cohort details: Table S1 and S2), approved by the Ethics Council of the Christian-Albrechts-University of Kiel, Germany (file number A422/07). An informed consent form was signed by all patients and/or their legal guardians. Paired peripheral blood mononuclear cells (PBMCs) samples were collected before treatment and on day 5 (after 4 r-hGH injections) and were subjected to transcriptome profiling using the Affymetrix Human Gene 1.0 ST microarray according to the manufacturer’s guidelines. This workflow mimics the routine IGF1-generation test. Monitoring PBMCs, which express a functional growth hormone receptor, reduces transcriptional noise from other cell types and decreases result variability. Patients were reassessed annually for up to 8 years, and relevant clinical parameters were recorded (chronological scheme: Fig. S1). To align with clinical scenarios, supervised linear support vector machine (SVM) models were trained to predict growth response from PBMCs transcriptomes sampled before and after treatment initiation. Response was defined as the cumulative height gain standard deviation score (SDS), a well-established measure of therapy response, at year 4. Of the 71 patients enrolled, 43 had available PBMCs transcriptomes and a complete 4-year follow-up, thus were eligible for prediction. The remaining participants were excluded due to reaching near-final height before year 4, family relocation, or inconsistent therapy adherence. Participants were categorized by cumulative height gain SDS after 4 years of therapy into high responders (upper third, *n* = 15), intermediate responders (middle third, *n* = 14), and low responders (lower third, *n* = 14). The models were initially trained to distinguish high versus low responders; intermediate responders were temporarily omitted to increase contrast and improve discrimination, then reintegrated to evaluate real-world performance. The cumulative height gain SDS is calculated employing age, sex, and height. Consequently, these parameters do not represent confounding factors.

The linear SVM approach used here is widely applied to predict treatment success and yields interpretable parameters that align with European and US artificial intelligence regulatory expectations. We corrected for multicollinearity by removing features that added no independent information. Predictive transcripts were then ranked via recursive feature elimination, and the resulting feature sets were evaluated in a training/test framework using 100 cross-validation cycles (2-fold repeated 50 times) to benchmark prediction accuracy (see Supplementary Material for details). In a similar setup, Stevens et al applied supervised machine learning to predict r-hGH therapy response up to 5 years^5^ and reported high accuracy using a larger set of growth-related genes from pre-treatment (basal) blood transcriptome data.^4^ Keeping this study in mind, we sought a substantially smaller predictive gene set without restricting feature selection to growth hormone-related genes.

Using this approach, we achieved a high median balanced accuracy with as few as five predictive transcripts. PBMCs collected at day 5 after 4 injections of r-hGH outperformed those from before therapy start samples: before treatment PBMCs achieved a median balanced accuracy of 0.92 (Fig. 1A), whereas day 5 PBMCs reached a median balanced accuracy of 1.0 (Fig. 1B), indicating perfect classification of all patients. Although most therapy response prediction studies rely on baseline data, our findings suggest that a challenged (after treatment) system is more informative for predicting treatment trajectories. The predictive gene sets differ substantially between before and after treatment samples, suggesting that r-hGH-induced processes better define treatment response. This conclusion is supported by previous studies,^2^^,^^3^ reporting r-hGH induced changes in mRNA expression that may serve as biomarkers. While baseline sampling remains desirable for therapy management, our results suggest that its role should be reevaluated. Although our study setup cannot determine causality for the 5 predictive transcripts identified in PBMCs after therapy, it is likely that these are interconnected with key regulators of cellular growth, such as the mTOR pathway.^5^ However, outlining such connections would require further mechanistic studies. Interestingly, functional links to growth hormone have been reported for 3 of the 5 transcripts, namely leucine-rich repeats and immunoglobulin-like domain protein (*LRIG3*), uridine diphosphate-N-acetylglucosamine pyrophosphorylase-1-like-1 (*UAP1L1*), and sperm protein associated with the nucleus on the X chromosome D (*SPANXE*). In contrast, no growth-hormone related role is known for maestro heat-like repeat family member 6 (*C8orf73*) and keratin-associated protein 20-2 (*KRTAP20-2*) (for further details on the 5 predictive transcripts, see Table S3). The model maintained high accuracy when all patients, including intermediate responders, were considered: an 88.4% prediction match documents robustness even when confronted with patients the linear SVM model had not encountered during training (Fig. 1C). Trained on diverse diagnoses, the model’s high accuracy appears independent of the underlying diagnoses (Fig. S2), which may reflect broadly similar growth hormone receptor expression and downstream signaling across these conditions. However, our setup does not allow us to conclude that our gene sets perform equally in all disease types addressed.

**Figure 1:**
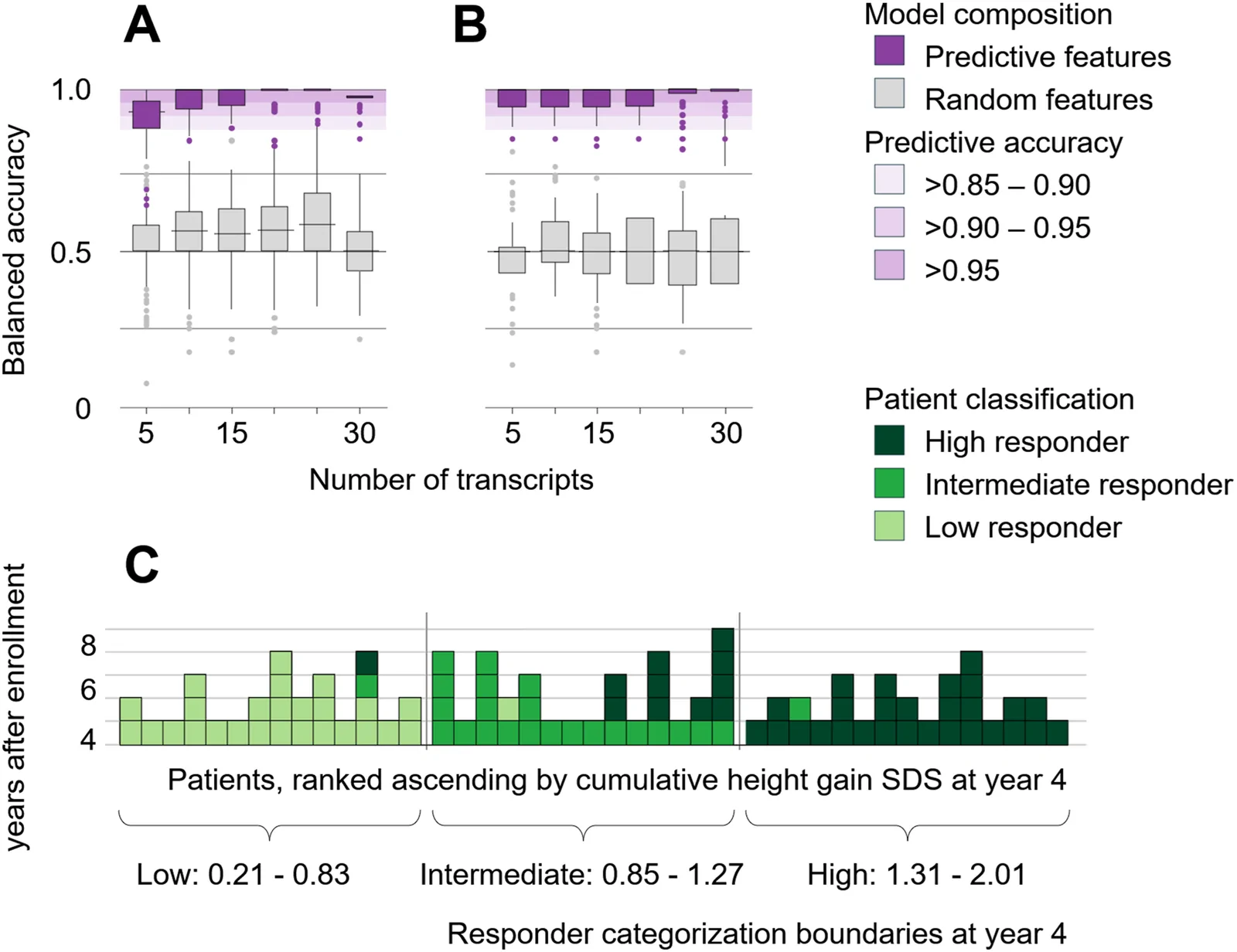
Balanced accuracy (*y*-axis) of individual linear support vector machine (SVM) models in relation to the number of transcripts included in the predictive sets. Transcript sets for the prediction are ranked (*x*-axis, left to right, 5–30) in the case of the predictive features (purple), while random features were not ranked (grey). Each boxplot represents 100 linear SVM models (median, boxes: 2nd and 3rd quartile, whiskers: data within 1.5 × IQR (interquartile range), dots: further outliers). **(A)** Transcriptome data measured at day 1 before therapy start (baseline). **(B)** Transcriptome data measured at day 5 after 4 consecutive injections of r-hGH (short term). **(C)** Schematic illustration of therapy responses for each patient (in columns) from year 4–8 (*y*-axis), ranked from left to right by cumulative height gain SDS at year 4. Boundaries frame the 3 response categories: low, intermediate and high responders. Color coding reflects the categorization at the corresponding year (4, 6 or 8). Upon discontinuation of the therapy, no further data are collected.

Of particular interest to clinicians is the long-term outlook beyond the 4-year prediction: our findings show that the 4-year prediction retains prognostic value for up to 8 years, with more than 70% of the patients remaining in the response category predicted at year 4 (Fig. 1C). This supports its utility for long-term therapy management. Our growth prediction model offers several advantages for potential clinical applications: 1) A panel of only 5 transcripts can be implemented in a cost-efficient, low-tech PCR setup. 2) Collecting blood after treatment initiation reveals responses at the molecular level, stabilizing expected trajectories. 3) Using PBMCs reduces transcriptional noise and thus lowers result variance. 4) Height gain SDS is an established outcome parameter within a clinically relevant 4-year observation period. 5) The predicted outcomes at year 4 retain prognostic value for up to 8 years. 6) Routine laboratory workflows yield results in 90–120 min without intensive labor. 7) The pre-trained model requires only quantitative PCR data, minimizing computational demands. 8) The costs per analysis are below 100 Euro. Although further validation in independent cohorts with larger sample sizes is desirable, particularly to confirm applicability across different diagnoses, no other monocyte-based cohort with a comparable 4-year follow-up setup is currently available. Keeping these limitations in mind, our prediction model addresses an unmet need in r-hGH therapy and offers a low-cost roadmap for improved long-term treatment with r-hGH.

## CRediT authorship contribution statement

**Valerie van Kekem:** Writing – original draft, Validation, Formal analysis, Data curation. **Irene Victoria Bermúdez-Pérez:** Writing – review & editing, Writing – original draft, Formal analysis, Data curation. **Alexandra E. Kulle:** Methodology, Formal analysis, Data curation. **Maik Welzel:** Methodology, Formal analysis, Data curation. **Nadine Hornig:** Methodology, Formal analysis, Data curation. **Gerhard Binder:** Writing – review & editing, Supervision. **Matthias Hübenthal:** Writing – review & editing, Supervision, Formal analysis, Data curation. **Robert Häsler:** Writing – review & editing, Writing – original draft, Visualization, Supervision, Methodology, Formal analysis, Data curation, Conceptualization. **Paul-Martin Holterhus:** Writing – review & editing, Writing – original draft, Supervision, Formal analysis, Data curation, Conceptualization.

## Funding

The study has been funded via a research grant by 10.13039/501100004191Novo Nordisk called: “Genome-wide analysis of rGH-dependent genes in peripheral blood monocytes for the identification of novel biomarkers of responsiveness to growth hormone therapy”.

## Conflict of interests

The authors declare that they have no conflict of interests. All authors have disclosed potential conflict of interests via the submission system, including financial support, advisory roles, and personal affiliations. The research conclusions remain independent.
