# Supplementary material for: Robust prediction of response to growth hormone therapy by five PBMC-based transcripts
Source: Genes Dis. 2026 Mar 6;14(1):102132. doi: 10.1016/j.gendis.2026.102132 (PMC13545363; doi:10.1016/j.gendis.2026.102132)
Supplement: Supplementary file 1 [file mmc1.docx]

**Supplementary Methods**

**Patients**

The study was designed as a single-centre study. It was approved by the Ethics Council of the Christian-Albrechts-University of Kiel, Germany (file number A422/07, April 4, 2008). An informed consent form was signed by all patients and/or their legal guardians. As part of this, a total of 71 patients (30 females, 41 males) were initially enrolled, based on defined endocrine diagnoses and proven formal indications for therapy with r-hGH established at the Hormone Centre for Children and Adolescents of the University Hospital of Schleswig-Holstein, UKSH, Kiel, Germany. The cohort included patients with growth hormone deficiency (n=49), bioinactive growth hormone (Kowarski syndrome, n=2), small for gestational age (n=16), and Turner syndrome (n=4). The median age at the start of therapy was 9.8 years (range 2.1 – 15 years). The median height SDS before therapy was -2.6 and the median growth rate SDS before therapy was -0.6 based on KiGGS reference percentiles (Kurth et al., “Design of the German KiGGS-Study” BMC Public Health, 2008).

**Sampling scheme of PBMCs at the start of therapy**

Two fasting morning routine blood samples were taken from each patient. One before the start of therapy on day 1 (baseline) and another after 4 days of treatment with 4 subcutaneous evening injections of 30µg/kg/day r-hGH (day 5, short-term, see Supplementary Fig. 1). This setting represents classical IGF1-generation test in which we earlier demonstrated transcriptional changes in PBMCs (Welzel et al., “Transcriptional response of peripheral blood mononuclear cells to recombinant human growth hormone in a routine four-days IGF-I generation test”, Growth Hormone & IGF Research 2011). 5 – 10 ml heparin whole blood was obtained at both time points for PBMCs isolation. In addition, IGF1 was determined at both time points by radioimmunoassay (RIA, Mediagnost, Reutlingen, Germany).

***Supplementary Figure 1****. Chronological scheme of blood sampling and follow-up examinations. Blood samples obtained on day 1 and day 5 were subjected to PBMC isolation and subsequent transcriptome analysis. r-hGH: recombinant growth hormone.*

**PBMC isolation**

PBMC isolation was performed as previously described (Welzel et al., “Transcriptional response of peripheral blood mononuclear cells to recombinant human growth hormone in a routine four-days IGF-I generation test”, Growth Hormone & IGF Research 2011) by Ficoll (Biochrom AG, Berlin, Germany) gradient centrifugation. Viability of PBMCs was checked by staining cells with a 1:10 dilution of Turk’s solution (Merck, Darmstadt, Germany). Collected PBMCs were resuspended in RPMI 1640 media (Gibco, Karlsruhe, Germany) with 10% DMSO, 10% heat-inactivated FCS, penicillin (100U/mL), streptomycin (100µg/mL), and L-glutamine (2 mM) and then immediately frozen in liquid nitrogen.

**RNA isolation and amplification**

RNA from PBMCs was isolated as previously described (Welzel et al., “Transcriptional response of peripheral blood mononuclear cells to recombinant human growth hormone in a routine four-days IGF-I generation test.”, Growth Hormone & IGF Research 2011). In brief, we applied the RNeasy Mini kit (Qiagen, Hilden, Germany). DNA impurities were eliminated using the DNA-free^TM^ kit (Ambion, Austin, USA). Total RNA quality and quantity were determined via a 2100 bioanalyzer (Agilent Technologies, Agilent, Palo Alto, USA). RNA-amplification was performed employing the MessageAmp II aRNA kit protocol (Ambion, Austin, USA) according to the manufacturer’s recommendations.

**Genome-wide transcriptome profiling**

Transcriptome profiling was performed using Affymetrix Human Gene 1.0 ST microarrays. RNA was processed as previously described (Mah et al., “A comparison of oligonucleotide and cDNA-based microarray systems”, Physiol Genomics, 2004), subsequently hybridized to the arrays and scanned following the manufacturer’s guidelines (Affymetrix, Thermofisher Scientific, Waltham, Massachusetts, USA). After normalization using the GCRMA method (R, Bioconductor), signals were considered present if their intensity exceeded two standard deviations above the median background (as determined by antigenomic control features on the array). The remaining features were subjected to downstream analysis for therapy outcome prediction.

**Clinical follow-up and selection of the 4 years study interval**

Patients were closely monitored during follow-up appointments every 3 months in the first year of r-hGH treatment and every six months thereafter by experienced pediatric endocrinologists at the Hormone Centre for Children and Adolescents at the University Hospital of Schleswig-Holstein, UKSH, Kiel, Germany. At each visit, key clinical parameters were assessed, including chronological age, height, (always using the same “Dr. Keller I” system, Längenmesstechnik GmbH Limbach, Limbach-Oberfrohna, Germany), height SDS, cumulative height gain SDS, growth velocity, growth velocity-SDS, BMI, BMI-SDS, pubertal status (per Tanner stage) and growth hormone dosage. R-hGH was administered subcutaneously every evening at home by the parents or by the older child if possible. After the first 4 injections of 30 µg/kg/d of r-hGH, the routine dose was adjusted to the diagnose-specific dose according to the legal approval.

The primary goal of predicting long-term therapy response had to be balanced with the steadily decreasing number of participants over time. Dropout reasons included reaching near-final height (leading to therapy cessation), families relocating during therapy, and irregular adherence to therapy protocols. For downstream analysis, the 4-year (long-term) observation was selected, during which 43 of the originally 71 enrolled patients remained on r-hGH treatment in our center.

**Supplementary Table 1. Cohort characteristics at the start of therapy**. Two patients with growth hormone deficiency and one patient with Turner syndrome were born small for gestational age.

| Diagnosis | n | Sex m/f | Age (years; median) | Height SDS (cm; median) | Bone Age (years; median) |
| --- | --- | --- | --- | --- | --- |
| Growth hormone deficiency | 49 | 28/21 | 10.1 | -2.6 | 8.8 |
| Kowarksi syndrome | 2 | 2/0 | 10.7 | -2.2 | 8.3 |
| Small for Gestational Age (SGA) | 16 | 11/5 | 9.3 | -2.8 | 6.4 |
| Turner syndrome (TS) | 4 | 0/4 | 6.1 | -2.4 | 6 |

**Supplementary Table 2. Clinical parameters in year 4 after treatment initiation**. One patient with growth hormone deficiency and one patient with Turner syndrome were born small for gestational age.

| Diagnosis | n | Sex m/f | Height SDS after 4 years  of therapy (range (median)) | Cumulative height gain SDS (range (median)) |
| --- | --- | --- | --- | --- |
| Growth hormone deficiency | 30 | 17/13 | -2.6 – -0.1 (-1.3) | 0.2 – 1.8 (1.2) |
| Kowarski Syndrome | 1 | 1/0 | -1.3 | 0.6 |
| Small for Gestational Age (SGA) | 9 | 8/1 | -2.1 – -0.4 (-1.6) | 0.7 – 2.0 (1.1) |
| Turner syndrome (TS) | 3 | 0/3 | -2.2 – -1.6 (-1.6) | 0.3 – 0.8 (0.7) |

# Support Vector Machine (SVM) modeling

- **Software and packages:** Linear SVM models were estimated using R (version 4.3.1) with the sigFeature (version 1.18.0), caret (version 6.0-94) and e1071 (version 1.7-13) packages.
- **Feature preprocessing:** Multicollinearity among predictive transcripts was reduced by excluding transcripts with a Spearman rank correlation >0.7.
- **Feature selection:** Recursive feature elimination (RFE) with sigFeature was performed once on the full dataset to obtain a ranked list of transcripts. The top 30 transcripts from this ranking were then used to construct the SVM models.
- **Benchmarking control:** A similar procedure was applied by drawing random features from the same set of transcripts to create a control for benchmarking predictive features.
- **Kernel and hyperparameter tuning:** Models were trained using a linear kernel (“C-classification”). Internal 5-fold cross-validation was performed for tuning of hyperparameter C∈{2^-5^, 2^-4^, ..., 2^5^}.

# Cross-validation

- **Setup:** Model performance was evaluated using repeated 2-fold cross-validation with 50 repeats, resulting in 100 training and test datasets.
- **Performance metrics:** Balanced accuracy (BA = (sensitivity + specificity)/2) was used as the primary metric.
- **Reporting:** Performance was summarized as the median BA across folds.

**Supplementary Results**

# Supplementary Table 3. Predictive transcripts and biological context

Top 5 transcripts, sorted by predictive rank generated by the linear SVM model applied to the short-term (day 5) data. Gene functions were retrieved from www.geneontology.org.

| Rank | Gene Symbol | Gene Name | Gene function |
| --- | --- | --- | --- |
| 1 | LRIG3 | Leucine-Rich Repeats and Immunoglobulin-Like Domains 3 | Involved in morphogenesis (otolith) |
| 2 | UAP1L1 | UDP-N-Acetylglucosamine Pyro phosphorylase 1 Like 1 | Involved in UDP-N-acetylglucosamine diphosphorylase activity and biosynthetic process |
| 3 | C8orf73 | Maestro Heat Like Repeat Family Member 6 | Not characterized, yet the data here shows a potential involvement in growth hormone response |
| 4 | SPANXE | Sperm Protein Associated with The Nucleus on The X Chromosome D | Not characterized, yet the data here shows a potential involvement in growth hormone response |
| 5 | KRTAP20-2 | Keratin Associated Protein 20-2 | Related to Keratinization and Nervous System development pathways |

***Supplementary Figure 2.*** *Principal component analysis,* *incorporating the top 5 transcripts, predictive for 4 years (long-term) outcome (dark and light green) using day 5 (short-term) observation samples. The initial patient diagnosis is represented in different shapes.*

# Data access

The transcriptome dataset employed in this study are available via GEO, accession number: GSE290942
